# Guided Metabolic Detoxification Program Supports Phase II Detoxification Enzymes and Antioxidant Balance in Healthy Participants

**DOI:** 10.3390/nu15092209

**Published:** 2023-05-06

**Authors:** Chinmayee Panda, Slavko Komarnytsky, Michelle Norton Fleming, Carissa Marsh, Keri Barron, Sara Le Brun-Blashka, Brandon Metzger

**Affiliations:** 1 Nutrition Innovation Center, Standard Process Inc., 150 N Research Campus Dr, Kannapolis, NC 28081, USA; kbarron@standardprocess.com (K.B.); slebrunblashka@standardprocess.com (S.L.B.-B.); bmetzger@standardprocess.com (B.M.); 2 Plants for Human Health Institute, NC State University, 600 Laureate Way, Kannapolis, NC 28081, USA; komarnytsky@ncsu.edu; 3 Department of Food, Bioprocessing and Nutrition Sciences, North Carolina State University, 400 Dan Allen Drive, Raleigh, NC 27695, USA; 4 College of Chiropractic, Northwestern Health Sciences University, 2501 W 84th Street, Bloomington, MN 55431, USA; mlnorton@nwhealth.edu (M.N.F.); cmarsh@nwhealth.edu (C.M.)

**Keywords:** whole food, botanical, detoxification program, liver, antioxidants, free radicals, hepatic markers, GST, SOD, GSH, GSH:GSSG ratio

## Abstract

Adequate antioxidant supply is essential for maintaining metabolic homeostasis and reducing oxidative stress during detoxification. The emerging evidence suggests that certain classes of phytonutrients can help support the detoxification process by stimulating the liver to produce detoxification enzymes or acting as antioxidants that neutralize the harmful effects of free radicals. This study was designed to examine the effects of a guided 28-day metabolic detoxification program in healthy adults. The participants were randomly assigned to consume a whole food, multi-ingredient supplement (*n* = 14, education and intervention) or control (*n* = 18, education and healthy meal) daily for the duration of the trial. The whole food supplement contained 37 g/serving of a proprietary, multicomponent nutritional blend in the form of a rehydratable shake. Program readiness was ensured at baseline using a validated self-perceived wellness score and a blood metabolic panel, indicating stable emotional and physical well-being in both groups. No significant changes or adverse effects were found on physical or emotional health, cellular glutathione (GSH) and the GSH:GSSG ratio, porphyrin, and hepatic detoxification biomarkers in urine. The intervention was positively associated with a 23% increase in superoxide dismutase (*p* = 0.06) and a 13% increase in glutathione S-transferase (*p* = 0.003) activities in the blood. This resulted in a 40% increase in the total cellular antioxidant capacity (*p* = 0.001) and a 13% decrease in reactive oxygen species (*p* = 0.002) in isolated PBMCs from participants in the detoxification group. Our findings indicate that consuming a whole food nutritional intervention as a part of the guided detoxification program supported phase II detoxification, in part, by promoting enhanced free radical scavenging and maintaining redox homeostasis under the body’s natural glutathione recycling capacity.

## 1. Introduction

The environment contains close to 80,000 novel chemicals, which are registered with the United States Environmental Protection Agency (EPA), but many have not had thorough reviews for their risk to human health [1]. Generally regarded as environmental toxins or pollutants, these chemicals belong to several identifiable groups, such as toxic elements (heavy metals), naturally occurring toxins (molds and their volatile metabolites, food allergens), pesticides, persistent organic pollutants, volatile organic compounds (solvents and fuels), and plastics [2]. As these compounds enter food chains and agricultural production systems [3], the assessment of the health risks associated with these chemicals is limited due to the lacking information on their long-term toxicity and exposure levels. Developmental and early years remain particularly susceptible to their effects [4].

Direct exposure, as well as the bioaccumulation of toxic chemicals in human tissues, make them a major continuous threat to human health. More so, the mechanisms by which environmental pollutants cause disruptive health effects may include tissue damage, endocrine signaling, genotoxicity, enzyme inhibition, oxidative stress, and changes in the activity of the gene expression networks that modulate them [5]. Severe pathological dysfunction may develop as a result once failure to maintain healthy levels of metabolic homeostasis [6], endocannabinoid signaling [7], immune responses [8], and tissue repair [9] are not timely resolved. Chronic exposure and improper clearance of environmental toxins, therefore, directly contribute to the development of obesity [10], cardiovascular disease [11], neurocognitive impairment [12], and reproductive concerns [13]. 

The human body largely relies on the liver to metabolize and direct exogenous chemicals to tissue storage or excretion [14]. This is achieved by the phase I enzyme-mediated oxidation or reduction of the toxic substrates, followed by the phase II enzymatic conjugation of the newly added or exposed functional groups to glucuronide, sulfate, or glutathione moieties. These conjugation reactions increase the water solubility of xenobiotics and tag them for carrier-mediated transport and excretion [15]. While the phase I reactions are mediated predominantly by the cytochrome P450 (CYP) superfamily of enzymes [16], the phase II system is more diverse and includes UDP-glucuronosyltransferases (UGTs), sulfotransferases (SULTs), methyltransferases (MTs), glutathione S-transferases (GSTs), and N-acetyltransferases (NATs) [17]. 

Additionally, the liver detoxification system is supported by endogenous antioxidants (GSH, reduced glutathione), dietary antioxidants (ascorbate), and antioxidant enzymes, such as superoxide dismutase (SOD), catalase, glutathione reductases, glutathione peroxidases (GPXs), and glutathione S-transferases (GSTs), that enable scavenging of reactive oxygen species (ROS) and free radicals arising from healthy cellular metabolism, as well as the detoxification of xenobiotics [18]. GSH is the most abundant endogenous antioxidant, which must be reduced and recycled from its inactive form (GSSG) in order to carry out additional antioxidant reactions [19]. The ratio of reduced to oxidized glutathione (GSH:GSSG) is an indicator of cellular health or cellular toxicity [20], as well as the redox status of the cell [21]. While there may be a need to repair low levels of glutathione, proper balance, rather than excess, is generally required [22]. 

Nutritional status and dietary intake patterns can have a profound effect on the body’s ability to absorb, conjugate, and excrete potentially dangerous toxins [1]. Certain botanically defined vegetable diets have been shown to enhance the hepatic conversion of xenobiotics [23] and support the body’s endogenous levels of glutathione [24]. A 12-week program combined with a 30-day dietary detoxification intervention (*n* = 12) resulted in significant changes to body composition as well as improved levels of zonulin and leptin [10]. Other clinical trials investigating strategies to enhance detoxification pathways exhibited high variability due to genetic and gender differences [25,26,27]. In this study, we focused on a cohort of healthy adults enrolled in a guided detoxification program that included a healthy diet education session with or without 28-day nutritional supplementation with a whole food, proprietary multicomponent blend. The primary objective was to quantify the functional markers of metabolic detoxification in blood and urine compared to the study baseline. The secondary outcomes were to determine the safety and tolerability of the intervention by assessing adverse event rates, comparisons of laboratory tests, and self-reported wellness data. 

## 2. Materials and Methods

### 2.1. Participants

**Figure 1:**
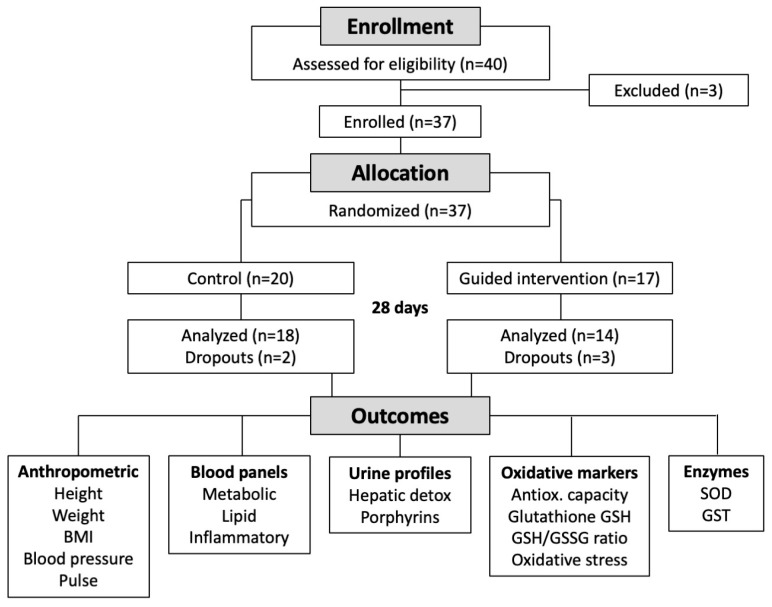
Flowchart of the study.

Flow of the participants through the study is shown in Figure 1. Using data generated previously [28], a sample size calculation with a *p* < 0.05 significance and 80% power revealed that 15–20 study participants would be needed to detect a 15% difference in the activity of detoxification enzymes in a randomized clinical study [29]. The study was a 4-week blinded, randomized controlled trial (RCT) with parallel assignment initiated in March of 2022 at Northwestern Health Sciences University’s De Rusha Clinic in Bloomington, Minnesota. The participants were heathy adults affiliated with the university who responded to an announcement regarding an upcoming lifestyle change program. The study staff completed an informational session that outlined the program and obtained health-related information from interested participants to determine study eligibility.

Forty potential participants responded to a posting. Thirty-seven were subsequently offered participation in the program and were randomized to serve as controls (a healthy diet education session alone) or to receive a healthy diet education session and a guided 28-day metabolic detoxification program, including the allocated intervention. The healthy diet education session included a PowerPoint presentation on healthy dietary guidelines and sample recipes of healthy meals. The guided component of the detoxification program included an additional PowerPoint presentation with the information about the investigational product, directions, and dosing information for its consumption. 

All healthy participants (29 female, 8 male, age range 20–60 years) met the inclusion criteria and provided informed consent. None had any exclusion criteria (Table 1). All participants completed the study procedures. Two control participants and three intervention participants were excluded from analyses due to study dropout. Females on birth control were excluded from the study. There were no protocol deviations. The investigators and outcome assessors were blinded to group allocation. Participant information and generated data were fully anonymized for data analysis and interpretation of results.

All research involving human participants was approved by the Advarra Institutional Review Board (IRB), protocol No. Pro00055058, and all clinical investigations were conducted according to the principles expressed in the Declaration of Helsinki. The study was registered on www.clinicaltrials.gov (accessed on 3 May 2023) (Identifier: NCT00000000). 

### 2.2. Study Investigational Product

The proprietary, whole food blend was supplied as 27.4 oz (777 g) SP Detox Balance bottles by the manufacturer (Standard Process Inc., Palmyra, WI, USA). The serving size was defined as 2 scoops (37 g) and included a blend of organic pea protein, flax meal, organic oat flour, organic pumpkin seed protein, organic buckwheat flour, organic beet (leaf) juice powder, organic buckwheat (aerial parts), apple pectin, juniper (berry) powder, organic Spanish black radish (root), burdock (root) powder, organic beet (root), calcium citrate, organic barley (grass), dandelion (leaf), broccoli (aerial parts), inositol, organic alfalfa (aerial parts) juice powder, Oregon grape (root) powder, globe artichoke (leaf), sunflower lecithin powder, milk thistle extract (80% silymarins), organic cordyceps mushroom powder, organic carrot, and organic sweet potato. Other ingredients included creatine, L-leucine, xanthan gum, L-isoleucine, L-valine, DL-methionine, monk fruit extract, and choline bitartrate. The nutritional intervention was self-administered by participants orally in the form of a rehydratable shake with 1–2 servings daily on days 1–7, 3 servings daily on days 8–21, and 1–2 servings on days 22–28. 

### 2.3. Anthropometrics 

Height, weight, BMI, blood pressure, and pulse were measured by the study staff on day 1 and day 28 of the study. Procedures were followed as per the Centers for Disease Control and Prevention guidelines. The standard Food Frequency Questionnaire (FFQ), the 24 h Dietary Recall Questionnaire (24 h), and the Medical Symptoms Questionnaire (MSQ) were administered to all participants at baseline and the end of the study to monitor study readiness, dietary patterns, and health-related events during the study. 

### 2.4. Laboratory Testing

Fasting blood samples were obtained at baseline (day 1) and on the last day of the study (day 28). The samples were collected in three BD Vacutainer SST tubes, allowed to clot at room temperature for 30–45 min. Then, serum was separated using centrifugation at 3400 rpm for 10 min and stored at −80 °C. Serum was analyzed for three panels of metabolic (ALT, ALK, AST, free T3, free T4, GGT, total T3, total T4, TSH, T-uptake, and Vitamin D), lipid (cholesterol, ultra HDL), and inflammatory (CRP, IgA, IgE, IgG, IgM) markers using the Abbott (Chicago, IL, USA) Architect ci4100 following the manufacturer’s instructions (Supplementary Table S1). 

Morning first void urine samples on day 1 and day 28 were collected for assessment of the hepatic detox profile (phase I D-glucaric acid, phase II mercapturic acids, and creatinine) and the urine porphyrin profile (uroporphyrins, heptacarboxylporphyrins, hexacarboxylporphyrins, pentacarboxylporphyrins, coproporphyrins 1-3, total porphyrins, including precoproporphyrins 1-3, total precoproporphyrins, and creatinine) using commercial testing services (Doctor’s Data, St. Charles, IL, USA). 

### 2.5. Antioxidant and Redox Status 

Total antioxidant capacity was measured as the combined antioxidant activities of all serum constituents, including vitamins, proteins, lipids, glutathione, and uric acid. The assay relied on the ability of antioxidants in the sample to inhibit the oxidation of ABTS (2,2′-Azino-di-[3-ethylbenzthiazoline sulphonate]) to ABTS•+ by metmyoglobin and was quantified as Trolox, a water-soluble tocopherol analogue, equivalents. The decrease in ABTS oxidation was quantified colorimetrically at 734 nm using a Biotek Synergy H1 spectrophotometer (Agilent, Santa Clara, CA, USA). 

Reduced glutathione (GSH), oxidized glutathione (GSSG), and total glutathione in serum samples were quantified in duplicate using fluorometric GSH/GSSG ratio detection assay kit (Abcam, Cambridge, MA, USA) at the excitation/emission of 490/520 nm (Biotek Synergy H1). 

### 2.6. Oxidative Stress 

Blood samples were collected in BD Vacutainer CPT mononuclear cell preparation tubes that contained blood separation media composed of a thixotropic polyester gel and a Ficoll Hypaque solution. The tubes were centrifuged to isolate live peripheral blood mononuclear cells (PBMCs), and oxidative stress was measured using a fluorogenic cell-permeant probe CellROX Orange (Thermo Fisher, Waltham, MA, USA). Fluorescence upon oxidation by reactive oxygen species was quantified on BD Accuri C6 flow cytometer with absorption/emission maxima of 545/565 nm and presented as relative fluorescent units (RFUs). 

### 2.7. Detoxification Enzyme Activity 

SOD activity was assessed using an assay that utilizes a tetrazolium salt for detection of superoxide radicals generated by xanthine oxidase and hypoxanthine using a superoxide dismutase assay kit colorimetrically at 450 nm (Cayman, Ann Arbor, MI, USA). One unit of SOD was defined as the amount of enzyme needed to exhibit 50% dismutation of the superoxide radical. 

Total GST activity was measured by quantifying the conjugation of 1-chloro-2,4-dinitrobenzene (CDNB) with reduced glutathione using a glutathione S-transferase assay kit colorimetrically at 340 nm (Cayman). 

### 2.8. Self-Reported Wellness 

Program readiness was ensured at baseline and the end of the study using self-reported wellness status as determined by PROMIS Global-10 questionnaire and a normal comprehensive blood metabolic panel result. The PROMIS Global short form was scored into a Global Physical Health component and Global Mental Health component. The summed raw scores from PROMIS Global were converted into standardized T-score distributions such that a 50 represents the average (mean) for the US general population [30].

### 2.9. Statistics 

Statistical analyses were performed using JMP 15.2.1 (SAS Institute, Cary, NC, USA). Datasets were tested for normality using the goodness-of-fit Shapiro–Wilks test. Descriptive statistics and two-tailed paired and unpaired Student’s *t*-test were used to evaluate changes in clinical outcomes at baseline and after intervention. All values were reported as mean ± standard error of mean (SEM), and statistical significance was set at *p* ≤ 0.05. Asterisks *, **, and *** indicate significance levels of *p* < 0.05, *p* < 0.01, and *p* < 0.001, respectively.

## 3. Results

### 3.1. Subjects’ Characteristics

A total of 37 participants were selected for the study and randomized into the control (*n* = 20, a healthy diet education session) or detox intervention groups (*n* = 17, a healthy diet education session followed by a guided 28-day metabolic detoxification program using the study investigational product). Three additional participants were screened but excluded from participating in the study based on medical conditions that did not qualify them further. 

Eighteen control and fourteen detox group participants completed the study as per the protocol (Figure 1). Two control and three detox group subjects were excluded from the study due to failing to comply with the study after the first visit (1); the remaining dropouts were excluded from the study based on voluntary withdrawal on the grounds of being unable to commit to the study protocol (4). The demographics and anthropometric data of the participants are shown in Table 2. While the detoxification group was able to maintain the parameters, including weight and BMI, throughout the study, control participants showed positive improvement in weight (*p* = 0.002) and BMI (*p* = 0.001) at the end of the study (Table 2). 

### 3.2. Safety and Tolerance Assessment 

No adverse effects attributable to the intervention were reported during the study. The laboratory safety endpoints included data from metabolic, lipid, and inflammatory panels and showed no significant changes (Supplementary Table S1). There were no significant observations in the Food Frequency Questionnaire (FFQ), the 24 h Dietary Recall Questionnaire (24 h), and the Medical Symptoms Questionnaire (MSQ) completed at baseline and the end of the study, suggesting that all participants followed the dietary education instructions provided to them at baseline and experienced no undesirable events for 4 consecutive weeks of the study.

### 3.3. Wellness Status and Quality of Life

**Figure 2:**
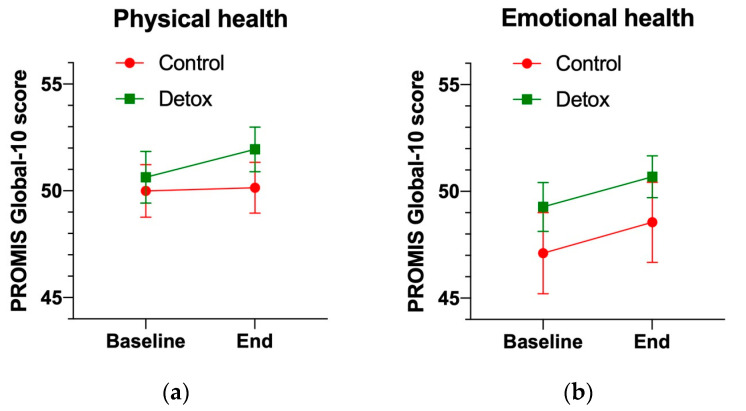
PROMIS Global Health-10 scores at baseline and at the end of the study, separated by (**a**) physical health and (**b**) emotional health components. Results are expressed as means ± SEM. Data were analyzed using the paired sample *t*-test.

Baseline physical and emotional health scores captured with the PROMIS Global-10 questionnaire were similar for both the control and detoxification groups and remained stable after the trial (Figure 2). The average unadjusted PROMIS Global Physical Health scores were 49.9 and 50.6 at baseline and 50.1 and 51.9 at the end of the study for participants in the control and intervention groups, respectively. Similarly, the average unadjusted PROMIS Global Mental Health scores were 47.1 and 49.3 at baseline and slightly increased to 50.1 and 51.9 at the end of the study.

### 3.4. Biomarkers of Oxidative Stress

**Figure 3:**
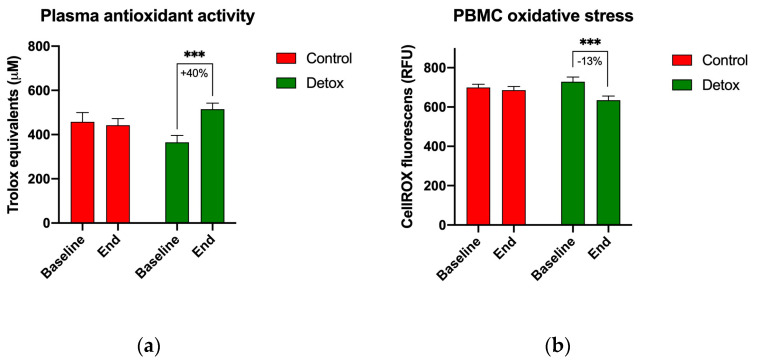
Changes in biomarkers of oxidative stress during the 4-week detoxification study. (**a**) Plasma antioxidant capacity measured using TEAC assay; (**b**) oxidative stress in the peripheral blood mononuclear cells measured using CellROX orange reagent. Results are expressed as means ± SEM. Data were analyzed using the paired sample *t*-test (*** *p* < 0.001).

At the baseline, the total plasma antioxidant capacity was measured as 457.9 μM and 365.2 μM for participants in the control and intervention groups, respectively. A 4-week detoxification program was associated with a 40% increase in total antioxidant capacity only in subjects receiving the intervention (514.7 μM, *p* < 0.001) (Figure 3a). This change correlated with an observed 13% decrease in ROS-associated oxidative stress in the peripheral blood mononuclear cells isolated from subjects receiving the intervention (724.4 RFUs at baseline versus 635.1 RFUs at the end of the study, *p* < 0.001) (Figure 3b).

### 3.5. Redox Homeostasis

**Figure 4:**
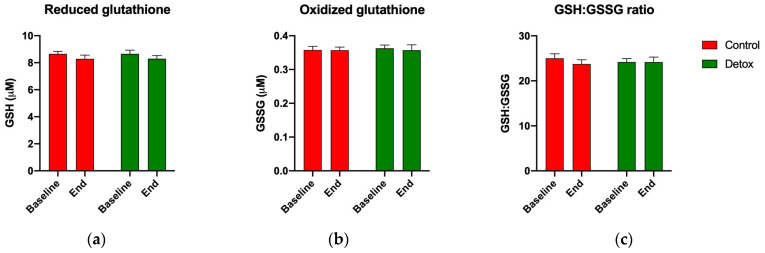
Changes in redox homeostasis during the 4-week detoxification study. (**a**) Reduced glutathione (GSH); (**b**) oxidized glutathione (GSSG); and (**c**) GSH/GSSG ratio as quantified using the fluorometric GSH/GSSG ratio detection assay. Results are expressed as means ± SEM. Data were analyzed using the paired sample *t*-test.

**Figure 5:**
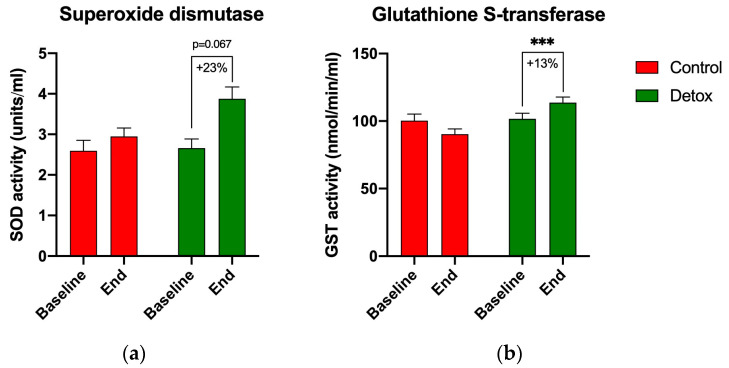
Changes in the activity of the phase II antioxidant enzymes during the 4-week detoxification study. (**a**) Superoxide dismutase (SOD); (**b**) glutathione S-transferases (GSTs) as quantified using the respective enzymatic assays. Results are expressed as means ± SEM. Data were analyzed using the paired sample *t*-test (*** *p* < 0.001).

Reduced glutathione (GSH) and the GSH:GSSG ratio remained stable in both groups (Figure 4), suggesting balanced redox homeostasis under the body’s natural glutathione recycling capacity. However, apparent changes in the activity of the phase II antioxidant enzymes superoxide dismutase (SOD) and glutathione S-transferases (GSTs) suggested an increased glutathione turnover in the participants receiving the detoxification intervention. The baseline SOD activity increased by 23% from 2.7 to 3.9 units/mL (*p* = 0.067) by the end of the study (Figure 5a), and the baseline GST activity increased by 13% from 101.6 to 113.7 nmol/min/mL (*p* < 0.001) for the detox participants only (Figure 5b). 

### 3.6. Detoxification Markers in Urine

Consistent with previous reports on high interindividual variation in the detoxification panels, we did not observe any significant reductions in the urine porphyrin biomarkers from both the control and detox cohorts (all *p*-values > 0.05)). A trend for increased urine D-glucaric acid (from 86.14 to 106.07 nM, *p* = 0.09) and decreased creatinine (from 103.50 to 91.50 mg/dL, *p* = 0.13) was observed only in the participants undergoing the detoxification treatment (Table 3).

## 4. Discussion

The human body is exposed to a lifetime variety of xenobiotics that need to be properly recognized, metabolized, and excreted to preserve and maintain health. Ubiquitous sources of xenobiotics include natural foods and drink products that are routinely digested and detoxified, as well as byproducts of their cellular metabolism that can build up over time and become harmful [31]. The development of synthetic chemistry, agrochemical, and pharmaceutical industries exponentially increased the amount of foreign chemical substances that must be recognized and neutralized [1]. Chronic exposure and improper clearance of these chemicals increase the body’s susceptibility to tissue injury and insufficient repair over time [5].

The bulk of tissue detoxification reactions occur in the liver and the gastrointestinal tract, with the kidneys, lungs, lymphatics, and skin tissues contributing to the different aspects of this process. Central to this process is adequate nutritional support for the activity of phase I (activation) [14] and phase II (conjugation) [15] enzymes, as these mechanisms exhibit significant individual variability affected by the environment, lifestyle, and genetic factors [32]. The use of plants and the consumption of plant-based foods have played a fundamental role in regulating the metabolism and elimination of xenobiotics, with probably the most famous example being grapefruit juice and its effects on the activity of hepatic detoxification enzymes [33]. Food structure, as it applies to whole foods, including texture- and matrix-related effects, also has direct effects on biomolecular availability and absorption in the gastrointestinal tract [34]. Additionally, the whole food matrix changes the availability of nutrients, such as amino acids or vitamins, that are required for many phase I and phase II detoxification reactions [35,36]. The increased intake of vegetables is protective of nonalcoholic fatty liver disease [37], hepatocellular carcinoma [38], and the overall risk of mortality from all causes [39]. These data justify and stimulate continuous research on a selection of whole food ingredients or formulations which are nutritionally complete, shelf–table, and hold promise to support tissue detoxification mechanisms. 

This randomized controlled study showed that a whole food, multi-ingredient nutritional intervention was well tolerated for 4 weeks with good palatability. The intervention showed no effect on general physiological outcomes, such as weight, BMI, blood pressure, and heart rate, in healthy volunteers, and did not affect the baseline values of the metabolic, lipid, and inflammatory panels. The main advantage of the tested intervention was the series of whole food ingredients used in its formulation. A mixture of pea protein, pumpkin seed protein, and oat flour was designed to deliver good levels of amino acids relevant to the detoxification reactions, including arginine (1300 mg), glycine (600 mg), isoleucine (850 mg), leucine (1600 mg), methionine (300 mg), and valine (900 mg) per serving. The total sugars (1 g) and total fats (5 g) were kept low using a mixture of flax meal, oat flour, and buckwheat flour. A series of functional ingredients was also selected to support the activity of the detoxification enzymes, including beets reported to activate glutathione S-transferase [40], Spanish black radish root for the activation of phase I/II enzymes [28], burdock root for reducing hepatotoxicity [41], apple pectin for its ability to detoxify heavy metals [42], juniper berries for their high antioxidant activity and ability to activate superoxide dismutase and glutathione peroxidase [43], dandelion leaf for protective effects against hepatic injuries [44], broccoli leaf for its capacity to modulate antioxidant and phase II enzymes, including SOD, via glucosinolate and isothiocyanate metabolism [45], globe artichoke leaf for the modulation of antioxidant and hepatic metabolism [46], milk thistle extract standardized to 80% silymarins to support healthy liver function [47], and cordyceps mushroom powder for its antioxidant and liver protection qualities [48]. 

The study also demonstrated that measurable biological effects on the total plasma antioxidant activity and oxidative stress in PBMCs can be achieved in healthy participants at intervention doses that are well tolerated. These findings correlated with increases in SOD and GST activities in the absence of the overall levels of reduced glutathione (GSH) or the GSH:GSSG ratio in the participating healthy volunteers. While multiple studies demonstrate the challenge associated with the maintenance of an endogenous glutathione pool, owing to a multitude of factors, including age and physiological and pathological conditions [49], proper balance, rather than excess, is generally required [22]. Many of the enzymes that participate in the antioxidant and glutathione recycling reactions require nutrient cofactors [50] that the current intervention was designed to supply. This may be responsible, in part, for the increased activities of SOD and GSTs, as many of the individual ingredients directly target or support these enzymes as discussed above. The ability of the intervention to increase phase II detoxification and antioxidant enzyme activity is in line with studies demonstrating the applicability of whole foods and certain phytonutrients to protect cells against electrophiles, DNA damage, and inflammation [51]. 

The current study also demonstrated high variability in hepatic detoxification (phase I D-glucaric acid, phase II mercapturic acids) and urine porphyrin profiles. The explanation for this intersubject variability is not obvious, however; individual responses to plant foods often depend on individual genetic polymorphisms and gender, as shown previously for GST enzymes [26] and cytochrome CYP enzyme families [52]. As the current intervention, similar to a regular healthy human diet, contains a wide variety of food ingredients, the precise individual effects of its supplementation on detoxification enzymes may be difficult to predict.

While this study offers novel insights into a whole food, nutritional intervention and its potential application to detoxification and antioxidant support, it does have notable limitations. We enrolled healthy volunteers, and it remains to be determined whether these findings apply generally to diverse patient populations with pre-existing disease states and metabolic pathologies. The majority of subjects were females, and this may limit our understanding of the effects of this supplementation in a male population. Furthermore, small but significant changes in BMI observed in the control population may be attributed to the placebo-like effects of the educational session and may be further explored in the future. This will be critical in determining the beneficial potential of the whole food, multi-ingredient supplementation in subsequent clinical studies in specific patient populations. 

In summary, this study both reports the successful development of a whole food, multi-ingredient nutritional intervention and demonstrates that its oral administration for 4 weeks improves the total plasma antioxidant activity and oxidative stress in PBMCs, in part, by increasing phase II detoxification and antioxidant enzyme activity, such as SOD and GSTs, in healthy participants. These effects were observed in the absence of apparent clinical side effects and changes in the laboratory biomarkers. Our work highlights the feasibility of translating the whole food, multi-ingredient supplementation and positions it as a novel formulation capable of supporting the body’s endogenous detoxification pathways. These results also present a strong case for incorporating functional biomarkers and guided education into personalized metabolic detoxification programs. 

## Figures and Tables

**Table 1:** Inclusion and exclusion criteria of the study.

Inclusion Criteria	Exclusion Criteria
Healthy adults in college settings aged 18–65 Willingness to comply with study protocol for 30 days No allergy to the ingredients in the study product Subject is a male or a nonpregnant, nonlactating female, at least 6 weeks postpartum prior to screening visit, and is not actively planning a pregnancy If on a chronic medication (that does not result in exclusion), subject has been on stable dose for at least two months prior to screening visit Subject has at least two-week wash-out period between completion of a previous research study that required ingestion of any study food or drug and their start in the current study	Subjects who are experiencing any adverse events due to any nutraceutical, OTC, pharmaceutical, or investigational products Celiac and other gastrointestinal health concerns Lipid lowering drugs or the use of anticoagulant medications in the preceding 4 weeks Pregnant and/or nursing Total cholesterol levels < 220 Subjects with untreated endocrine, neurological, skin or infectious diseases Significant liver or kidney disease Conditions affecting joint health Serious medical illness, including cancer Use of ethanol within 24 h of the evaluation visits (baseline, 4 weeks)

**Table 2:** Demographic and anthropometric data of the study participants (±SEM).

	Control (*n* = 18)		Detox (*n* = 14)	
	Baseline	End of Study	Baseline	End of Study
Age (years)	33.6 ± 3.2		36.9 ± 3.2	
Gender (male/female, %)	5/13 (27.8%)		2/12 (14.3%)	
Height, cm	167.75 ± 3.02	167.75 ± 3.02	163.78 ± 2.90	163.79 ± 2.90
Weight, kg	80.69 ± 4.66	80.33 ± 4.70 ** (*p* = 0.002)	70.59 ± 5.29	70.52 ± 5.33
BMI, kg/m^2^	27.04 ± 1.52	26.58 ± 1.48 *** (*p* = 0.001)	26.72 ± 1.66	26.63 ± 1.64
Systolic BP, mmHg	123.94 ± 3.03	120.33 ± 3.90	117.21 ± 2.43	118.14 ± 5.15
Diastolic BP, mmHg	79.67 ± 2.65	78.72 ± 3.13	74.07 ± 1.45	71.93 ± 1.73
Pulse, bpm	69.75 ± 2.77	69.75 ± 2.70	66.43 ± 2.17	66.00 ± 1.55

Results are expressed as means ± SEM (** *p* < 0.01, *** *p* < 0.001).

**Table 3:** Hepatic detoxification markers in urine (±SEM).

	Reference Range	Control			Detox		
		Baseline	End of Study	*p*	Baseline	End of Study	*p*
D-glucaric acid, phase I (nM/mg)	40–400	104.67 ± 12.62	122.89 ± 20.23	0.35	86.14 ± 8.03	106.07 ± 3.82	0.09
Mercapturic acids, phase II (μM/mM)	40–95	48.56 ± 2.34	48.61 ± 2.34	0.98	55.14 ± 3.82	51.07 ± 3.28	0.38
Creatinine, mg/dL	40–325	101.00 ± 10.12	119.33 ± 17.80	0.33	103.50 ± 11.48	91.50 ± 9.30	0.13

## Data Availability

The data presented in this study are available on reasonable request from the corresponding author.

## Supplementary Materials

The following supporting information can be downloaded at: https://www.mdpi.com/article/10.3390/nu15092209/s1, Supplementary Table S1: Metabolic, lipid, and inflammatory panel data (±SEM).
