# Supplementary material for: Guided Metabolic Detoxification Program Supports Phase II Detoxification Enzymes and Antioxidant Balance in Healthy Participants
Source: Nutrients. 2023 May 6;15(9):2209. doi: 10.3390/nu15092209 (PMC10181083; doi:10.3390/nu15092209)
Supplement: Supplementary file 1 [file nutrients-15-02209-s001.zip › nutrients-2370399-supplementary.pdf]

nutrients

MDPI

Supplementary Table S1. Metabolic, lipid, and inflammatory panels data (± SEM).

	Reference range	Control			Detox		
		**Baseline**	**End of study**	**P**	**Baseline**	**End of study**	**P**
Alanine aminotransferase, U/L	4-36	24.33 ± 3.83	17.44 ± 1.76	0.11	16.14 ± 1.14	17.64 ± 1.63	0.46
Alkaline phosphatase, U/L	44-147	67.67 ± 5.50	64.89 ± 5.15	0.71	62.78 ± 4.26	61.86 ± 4.98	0.89
Aspartate aminotransferase, U/L	8-33	21.11 ± 1.59	18.11 ± 0.95	0.11	19.14 ± 1.03	17.93 ± 1.14	0.43
Cholesterol, mg/dl	<200	196.00 ± 7.95	180.00 ± 7.98	0.16	199.20 ± 11.38	179.36 ± 12.46	0.25
C-reactive protein, mg/dl	<1	2.73 ± 1.26	1.41 ± 30.38	0.32	1.72 ± 0.62	1.52 ± 0.47	0.79
Gamma-glutamyl transferase, U/L	5-40	20.06 ± 2.43	16.22 ± 1.11	0.16	17.85 ± 2.20	16.00 ± 2.01	0.54
HDL Ultra, mg/dl	>45	57.22 ± 4.13	54.94 ± 3.80	0.69	61.21 ± 4.49	56.07 ± 4.56	0.43
Immunoglobulin A, mg/dl	61-356	175.83 ± 11.08	175.56 ± 11.18	0.99	169.00 ± 16.20	163.71 ± 18.92	0.83
Immunoglobulin E, mg/dl	1.5-144	137.96 ± 32.99	129.71 ± 31.30	0.90	130.00 ± 38.76	128.95 ± 37.45	0.99
Immunoglobulin G, mg/dl	700-1600	1066.39 ± 67.93	1105.06 ± 66.09	0.69	1021.40 ± 62.30	996.71 ± 78.25	0.81
Immunoglobulin M, mg/dl	46-152	128.78 ± 13.89	129.78 ± 16.24	0.96	111.60 ± 14.39	108.07 ± 15.53	0.87
Free T3, pg/ml	2-4.4	2.97 ± 0.09	2.93 ± 0.06	0.70	2.86 ± 0.09	2.65 ± 0.11	0.15
Free T4, ng/dl	0.9-2.3	0.95 ± 0.02	0.97 ± 0.01	0.46	0.93 ± 0.03	0.93 ± 0.03	0.92
Total T3, ng/ml	0.8-2	0.98 ± 0.03	0.93 ± 0.03	0.28	0.96 ± 0.04	0.87 ± 0.05	0.14
Total T4, ug/dl	5-12	6.82 ± 0.21	7.00 ± 0.22	0.55	6.66 ± 0.31	6.49 ± 0.31	0.69
TSH, uIU/ml	0.5-5	2.77 ± 0.55	2.43 ± 0.50	0.65	3.10 ± 0.92	3.15 ± 0.94	0.97
T-Uptake, units	0.7-1.2	0.98 ± 0.03	0.97 ± 0.02	0.79	0.95 ± 0.02	0.93 ± 0.03	0.44
Vitamin D, ng/ml	20-40	33.68 ± 4.60	33.52 ± 4.73	0.98	38.50 ± 5.78	33.14 ± 3.92	0.45
